# Interarm Difference in Blood Pressure, an Unspoken Necessity: A Comparative Study Among Normotensive and Age-Matched Hypertensive Individuals in a Tertiary Care Centre in South India

**DOI:** 10.7759/cureus.101999

**Published:** 2026-01-21

**Authors:** Arathi P Nair, Deepa Sivaraj, Saraswathy L, Renjitha Bhaskaran

**Affiliations:** 1 Department of Physiology, Amrita Institute of Medical Sciences, Amrita Vishwa Vidyapeetham, Kochi, IND; 2 Department of Biostatistics, Amrita Institute of Medical Sciences, Amrita Vishwa Vidyapeetham, Kochi, IND

**Keywords:** blood pressure, diastolic, hypertension, interarm difference, mean arterial pressure, systolic

## Abstract

Background and objective: Blood pressure (BP) is an important component of clinical assessment. Hypertensive management guidelines have instructed healthcare professionals to measure BP in both arms. Due to time constraints, BP is usually checked only in one arm. This study focuses on the prevalence as well as the comparison of interarm difference (IAD) in BP among different age groups and in individuals with hypertension.

Methods: A cross-sectional analytical study was conducted on 159 subjects in a tertiary care centre in South India. The BP was recorded in both arms sequentially within a short interval.

Result: The systolic IAD was found to be greater than or equal to 10 in 7.5% of normotensives in the younger age group, 13.2% of normotensives in the older age group, and 26.41% of hypertensives. The diastolic IAD was greater than or equal to 7 among 17% of normotensives in the younger age group, 15.1% of normotensives belonging to the older age group, and 26.4% of hypertensives.

Conclusion: A clinically meaningful difference in interarm BP was observed; however, due to the limitation caused by our small sample size, this difference did not reach statistical significance on comparison of IAD among these individuals (p > 0.05). This should not take away from the clinical relevance provided by the results. The results of this study underline the importance of checking BP in both arms and the need to make it a part of daily clinical practice.

## Introduction

Blood pressure (BP) is defined as the force exerted by blood against any unit area of the vessel wall. If a person's BP is 100 mm Hg, it means that the force is sufficient to push a column of mercury against gravity to a height of 100 mm [1]. Several studies support and emphasise the importance of measuring BP in both arms. Under normal physiological circumstances, BP readings are almost the same in both arms. However, a difference in BP between the arms is frequently encountered. This phenomenon is referred to as the interarm difference (IAD). Detecting an interarm BP difference is vital for further vascular assessment and the management of risk factors [2]. Knowledge regarding its prevalence and importance in accurate BP measurement remains poor, representing a potential source of error [3].

Hypertension is both a disease and a major risk factor for other diseases [4]. The presence of IAD in BP can make the diagnosis of hypertension difficult. A meta-analysis by Verberk et al. obtained the mean absolute IAD as 5.4 ± 1.7 and 3.6 ± 1.2 mm Hg for systolic and diastolic BP, respectively. Of the subjects studied, 14% had a systolic IAD ≥10 mmHg, 4% a systolic IAD ≥20 mmHg, and 7% a diastolic IAD ≥10 mmHg [5]. The study by Fotherby et al. compared the IAD in young and elderly individuals and noted that the range of interarm BP differences was wide [6]. 

Weber et al., in their study, showed that few participants had a BP > 140/90 mm Hg in one arm but not in the other, suggesting they could be denied referral for further investigation to diagnose hypertension against this threshold if BP was only measured in one arm [7]. Studies show how population-specific variation in the prevalence of IAD should be taken into account in delivering clinical care and in planning future studies [8]. 

There are very few studies on this topic that have been conducted in India. Most of the studies are prevalence studies, which focus on elderly individuals in Western countries. To our knowledge, there have been no studies on age group-based comparisons of IAD in BP. Moreover, there is limited data regarding the variability in IAD of normal individuals and hypertensive individuals. The primary objective of this study is to establish the prevalence of IAD in BP among different age groups and age-matched hypertensive individuals. The secondary objective is to compare IAD in BP among the aforementioned groups.

## Materials and methods

This is a cross-sectional comparative study conducted at a tertiary care centre in South India. The sample size was based on the mean and SD observed in a small pilot study featuring 10 individuals belonging to the younger age group and 10 belonging to the older age group. This pilot study was conducted six months before the main study in the same setting to get an appropriate and accurate sample size for our study population. 

A total of 159 participants were included in the current study and split into three groups of 53. Group I featured normotensive individuals aged 18 to 40 years, group II had normotensive individuals aged 48 to 70 years, and group III included hypertensive individuals aged 48 to 70 years. We excluded the 40 to 48 age group to avoid selection bias and selected the age category of 18 to 40 for the younger participants and 48 to 70 for the older participants to get the same range.

The Ethics Committee of Amrita School of Medicine (Kochi, KL, IND) issued approval in 2023 (approval no. ECASM-AIMS-2023-398). The study participants were mostly students and patient bystanders for groups I and II. Hypertensive individuals were selected from patients who came to the OPD or were admitted to the general medicine ward and who were previously clinically detected as hypertensive. The participants who were known cases of cardiac abnormalities, renal disorders, and peripheral vascular occlusive disease were excluded from the study. Informed consent was taken from all the participants. 

Blood pressure in both arms of these participants was taken using an Omron automatic BP monitor (Omron Healthcare Co. Ltd., Kyoto, JPN). The participants were told to relax for five to 10 minutes, and then their BP was recorded. The BP of both arms was recorded sequentially by the same observer within a short interval. The difference between these two values gave us IAD I. Five minutes later, the procedure was repeated to obtain IAD II. The average of both these values gave us the final IAD. The systolic IAD cutoff value was taken as 10 mmHg based on the IAD study done in Kancheepuram [2], but the diastolic IAD cutoff value was taken to be 7 mmHg in view of diastolic BP values being lower than that of systolic.

We also wanted to understand the IAD in mean arterial pressure (MAP). The MAP is not the arithmetic mean per se, as the duration of diastole is longer than that of systole. So the diastolic pressure (DP) is more significant in determining the MAP. The MAP of each arm was calculated using the formula of DP + ⅓ pulse pressure (PP), which is the difference between the systolic and diastolic pressure. The difference between the right and left MAP was taken, and a value of 10 or more was considered significant.

Statistical analysis was performed using SPSS Statistics version 20.0 (IBM Corp., Armonk, NY, USA). Categorical variables were expressed as frequency and percentage. Continuous variables were presented by median (Q1-Q3). To test the statistical significance of the difference in the median of continuous variables with gender and age class, the Mann-Whitney U test was used. To test the statistical significance of the agreement of different variables between the right and left sides, the intraclass correlation (ICC) coefficient was computed. To test the statistical significance of the difference in IAD categorization between groups, the chi-square test was used. A p-value of less than 0.05 was considered significant.

## Results

**Figure 1:**
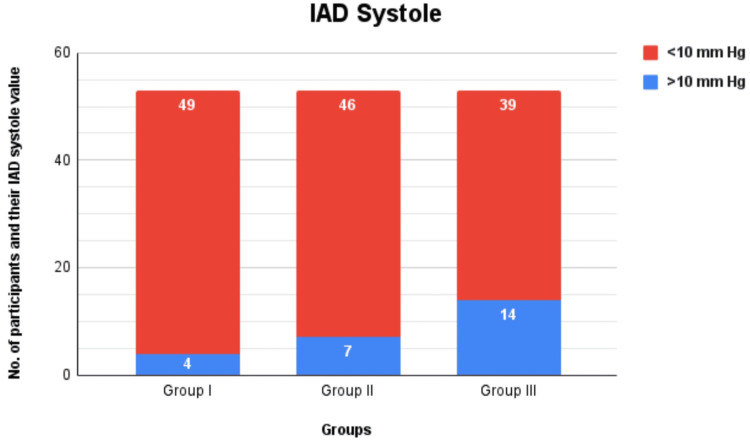
Comparison of IAD systole among different groups IAD: Interarm difference

A total of 159 participants were included in this study and split into three groups, each containing 53 participants. There were 82 female participants and 77 male participants distributed among the three groups. We found that the systolic IAD was ≥10 in 7.5% of participants of group I (four out of 53), 13.2% of group II (seven out of 53), and 26.41% of group III (14 out of 53) (Figure 1). No statistical significance was found between IAD systole in group I and group II. The p-value recorded on comparing the IAD systole of group II and group III (p = 0.088) is closer to the statistically significant p-value of 0.05 (Table 1). The maximum systolic IAD was recorded to be 18 in group I (a female participant) and group II (two female participants), and 24 in group III (a male participant). The median systolic IAD was found to be 4 (2-6) in group I and group II and 4 (2-10) in group III.

**Table 1:** Comparison of IAD systole among different groups *Chi-square test; a p-value of less than 0.05 is considered significant; IAD: Interarm difference

Group	Group I (n = 53)	Group II (n = 53)	Chi-square value	p-value	Group II (n = 53)	Group III (n = 53)	Chi-square value	p-value
IAD systole	4 (7.5%)	7 (13.2%)	0.913	0.526	7 (13.2%)	14 (26.4%)	2.91	0.088

**Figure 2:**
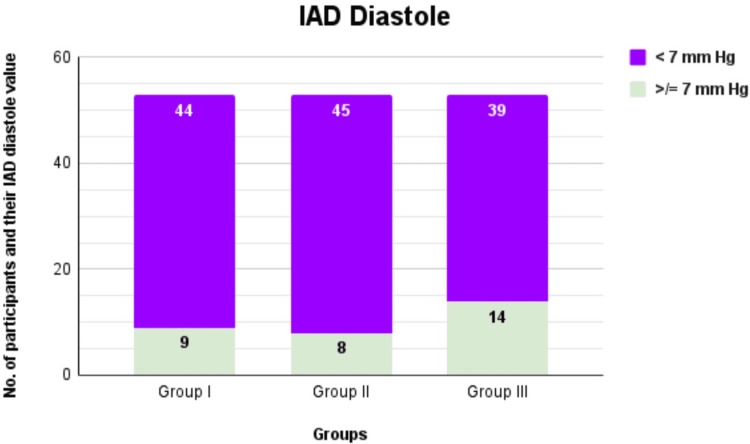
Comparison of IAD diastole among different groups IAD: Interarm difference

The diastolic IAD was ≥7 among 17% of group I participants (nine out of 53), 15.1% of group II (eight out of 53), and 26.4% of group III (14 out of 53) (Figure 2). No statistical significance was noted between the groups (Table 2). The maximum diastolic IAD was recorded to be 12 in group I (a male participant) and 16 for groups II and III (values were obtained in female participants). The median diastolic IAD was found to be 4 (1-6) in group I, 4 (2-6) in group II, and 3 (2-7) in group III.

**Table 2:** Comparison of IAD diastole among different groups *Chi-square test; a p-value of less than 0.05 is considered significant; IAD: Interarm difference

Variable	Group I (n = 53)	Group II (n = 53)	Chi-square value	p-value	Group II (n =53)	Group III (n = 53)	Chi-square value	p-value
IAD diastole	9 (17%)	8 (15.1%)	0.07	0.791	8 (15.1%)	14 (26.4%)	2.065	0.151

The median (Q1-Q3) of systolic IAD was found to be 3 (2-7) in females and 4 (2.5-8) in males. The median systolic IAD was higher among males. This was found to be statistically significant (p = 0.043) (Table 3). 

**Table 3:** Comparison of IAD systole among male and female participants *Mann-Whitney U test; a p-value of less than 0.05 is considered significant; IAD: Interarm difference

IAD systole	Median (Q1-Q3)	p-value
Female	3 (2-7)	0.043*
Male	4 (2.5-8)	

Of the 25 participants who had high systolic IAD, 14 were men (56%). The diastolic IAD was ≥7 in 31 participants; 18 of them were male (58.06%). Thus, the prevalence of systolic and diastolic IAD being ≥10 and 7, respectively, was higher in men. 

The MAP was calculated for both arms. The difference between right and left arm MAP was calculated, and 6.9% of individuals (11 out of 159) had a difference ≥10. Of these 11, only one participant from group I (1.8%), four from group II (7.5%), and six from group III (11.3%) showed this finding. The maximum IAD of MAP was recorded to be 10 in group I, 12 in group II, and 14 in group III. 

The systolic BP and diastolic BP readings were higher in the left arm in 85 and 83 participants, respectively. But the right and left arm readings showed good agreement with each other (ICC = 0.876 and ICC = 0.781, respectively). Thus, the ICC coefficient of systolic BP was higher than that of diastolic BP (Table 4). There was no statistically significant difference in the median systolic and diastolic IAD between group 1 and group 2 and between group 2 and group 3 (Tables 5-6).

**Table 4:** The ICC coefficient of systolic and diastolic BP *The systole ICC is greater than the diastole ICC. Therefore, the chance of there being a significant IAD in diastolic BP is higher when compared with systolic BP. ICC: Intraclass correlation, BP: Blood pressure, IAD: Interarm difference

Variable	ICC (95% CI)
Systolic BP (right arm-left arm)	0.876*
Diastolic BP (right arm-left arm)	0.781*

**Table 5:** Comparison of IAD systole and diastole among different age groups *Mann-Whitney U test; a p-value of less than 0.05 is considered significant; IAD: Interarm difference

IAD	Group	Median (Q1-Q3)	p-value
IAD systole	I	4 (2-6)	0.806*
	II	4 (2-6)	
IAD diastole	I	4 (1-6)	0.838*
	II	4 (2-6)	

**Table 6:** Comparison of IAD systole and diastole among normotensive and hypertensive individuals *Mann-Whitney U test; a p-value of less than 0.05 is considered significant; IAD: Interarm difference

IAD	Group	Median (Q1-Q3)	p-value
IAD systole	II	4 (2-6)	0.125*
	III	4 (2-10)	
IAD diastole	II	4 (2-6)	0.416*
	III	3 (2-7)	

## Discussion

Our study aimed to assess interarm BP differences in normotensive individuals aged 18 to 40 and 48 to 70 years, as well as in hypertensive individuals. An interarm systolic difference ≥10 mmHg was observed in 26.4% of hypertensive participants, demonstrating clinical significance. In comparison, a meta-analysis by Clark et al. reported a prevalence of 11.2% for systolic IAD ≥10 mmHg among hypertensive individuals (95% CI: 9.1-13.6; n = 3858) [8]. The borderline statistical significance in our study may be attributed to the smaller sample size (n = 159; 53 per group).

In our study, the maximum systolic IAD of 24 mmHg was observed in a hypertensive male, while the maximum in other groups was 18 mmHg. A systolic IAD ≥15 mmHg is recognized as an indicator of increased vascular disease and mortality risk, as reported in another meta-analysis by Clark et al. [9]. This finding warrants further evaluation of these individuals, as the 2018 European Society of Cardiology/European Society of Hypertension (ESC/ESH) guidelines also associate a systolic IAD ≥15 mmHg with an increased risk of cardiovascular events [10].

Another study consistent with our findings is the systolic BP difference between arms and cardiovascular disease in the Framingham Heart Study, conducted with 3,390 participants who were above the age of 40; the participants with an elevated interarm systolic BP difference were older (63.0 years vs. 60.9 years) [11]. Another cross-sectional study carried out in the Sulaimanyah governorate in the Kurdistan Region of Iraq, among 3030 young and healthy participants, found the prevalence of high systolic IAD to be the same as in our study (26%), but high diastolic IAD was found to be higher (30.5%) [12]. In our study, we found the median IAD systolic to be 4 (2-6) in group I and group II and 4 (2-10) in group III. In a study done to assess the IAD in BP among 462 participants, the systolic BP mean difference between the right and left arms was 1.1 mmHg [13]

Compared with the other groups, a higher diastolic IAD (≥7 mmHg) was predominantly observed in the hypertensive group (26.4%). In contrast, previous studies have defined elevated diastolic IAD as >10 mmHg. In Lane et al.'s study, 400 participants reported diastolic IAD of >10 and >20 mmHg in 11% and 3.5% of participants, respectively [14], while a cohort study among hypertensive individuals reported a prevalence of 6% for diastolic IAD >10 mmHg [15]. The higher prevalence observed in our study may be attributed to the lower cutoff value (≥7 mmHg) used to define elevated diastolic IAD.

In our study, the maximum IAD diastolic was recorded to be 16 in two female participants belonging to the older normotensive and hypertensive group. This warrants the need for further evaluation, as values of IAD diastolic higher than 10 are associated with increased risk of cardiovascular mortality, a fact that was highlighted by the interarm BP difference and mortality in patients with acute ischemic stroke study [16]. This study also showcased that the presence of an interarm systolic or diastolic BP difference ≥10 mmHg is a strong independent prognostic marker in acute ischemic stroke.

Higher IAD systolic and diastolic pressures are not only associated with cardiovascular mortality but also interfere with the general well-being of the patient. This finding was highlighted by a study conducted among rural elderly residents in the Guizhou Province of China [17]. This study identified that the incidence of mild cognitive impairment was higher among those elderly participants who had an IAD systolic and diastolic pressure >10. This further cements the need for recording BP readings in both arms and making it a mandatory part of our clinical examination. 

Through our study, we found a statistically significant relation between gender and systolic IAD, as the median IAD systolic was higher among males (p = 0.043). This was a clinically significant finding as well (56% of the participants who had a higher IAD were men). A clinically significant relation was also established between higher IAD diastolic value and men, as nearly 58.6% of the participants who had high IAD diastolic values were men. This was in disparity with Dahl et al.'s study, where the IAD was more predominant in women (26.8%) than men (21.0%) (p < 0.001) [18]. This study included 2,220 women and 2,297 men. Our findings are in accordance with the prevalence of the interarm BP difference study done among young healthy individuals in Iraq, where male participants were shown to have a higher IAD (p < 0.001) [12]. Studies have shown that women are more protected from hypertension and cardiovascular morbidity due to the influence of estrogen during the reproductive age [19]. This might be the reason why in our study, men have a higher prevalence of higher systolic and diastolic IAD.

We also found that the prevalence of high systolic and diastolic IAD was higher among the hypertensive group (26.4%). These findings are similar to those of Essa et al.'s where the association of high IAD was found not just with hypertension but also with smoking [12]. Yoon et al. showed that the systolic IAD level of the metabolic syndrome group (9.3±0.7 mmHg) was significantly higher (p <0.001) than for the non-metabolic syndrome group (5.7±0.3 mm Hg) [20]. Hypertension is one of the parameters used in the diagnosis of metabolic syndrome. Kimura et al.'s study conducted in Japan to assess the factors associated with IAD in BP also found a significant increase in systolic IAD >10 among hypertensive individuals [21]. 

In our study, the systolic and diastolic BP reading was higher in the left arm. Another study done to assess the consistency in interarm BP difference noted that the BP in the right arm tends to be higher than that of the left arm [22]. The aforementioned study also noted that the interarm BP difference was consistent only when obstructive arterial disease was present. This could have been the reason why the right and left arm readings in our study were in agreement with each other (ICC = 0.876 and ICC = 0.781 for systolic and diastolic BP, respectively). The ICC of systolic BP is higher than that of diastolic pressure. This implies that the chance of there being a significant IAD in diastolic BP is higher when compared to systolic BP. Hence, the diastolic reading should also be noted carefully in both arms. 

Even though a clinically significant difference in prevalence was found in the IAD systolic and diastolic with respect to age and hypertension, a statistically significant difference was not determined. This can be accounted for by the lower sample size of our study (total n = 159). Lane et al. in their study ascertained that age was the only significant predictor of clinically significant variations in interarm BP and mean absolute BP differences [14]. 

Additionally, our study also found the mean arterial pressure difference between the arms. This was not featured by any of the previous studies mentioned above. The MAP is an important tool used in clinical care. A meta-analysis to determine the accuracy of MAP as a predictor of pre-eclampsia found that mean arterial pressure is a better predictor of preeclampsia than systolic BP, diastolic BP, or an increase in BP [23]. Many studies have also shown MAP to be an important prognostic marker in septic shock [24]. As expected, the high MAP IAD was also found to be in higher prevalence among the hypertensive participants. 

One of the shortcomings of this study is the smaller sample size compared to other studies that have been referenced. This should not take away from the clinically suggestive nature of our findings, which were obtained after analyzing the IAD values among the three groups. Due to time and logistical constraints, we could only record the BP of both arms sequentially rather than simultaneously. The study results would have also been further enhanced if individuals with varying cardiovascular comorbidities were included. The IAD values obtained from these individuals would have further driven home the message of measuring BP in both arms. 

## Conclusions

Our study emphasizes the importance of measuring BP in both arms in all routine clinical examinations. It is of more significance in the follow-up care of hypertensive individuals as well as in other cardiovascular disorders. Our study highlights the need for a thorough follow-up for patients in whom systolic IAD and diastolic IAD are significantly higher, as it may be suggestive of cardiovascular morbidity, cognitive impairment, or cerebrovascular accident. The presence of significant IAD in BP measurement may be considered an indicator for these ailments to avoid future ramifications. 
